# Left Behind After Impact: The Hidden Toll of Trauma Recovery in New Zealand

**DOI:** 10.1111/ans.70722

**Published:** 2026-05-05

**Authors:** Sarah Logan, Duncan Finlayson, Nikita Quinn, Daniel Jemberie, Laura R. Joyce, Andrew McCombie, Christopher Wakeman

**Affiliations:** ^1^ Department of Surgery Te Whatu Ora Waitaha Canterbury Christchurch New Zealand; ^2^ Emergency Department Te Whatu Ora Waitaha Canterbury Christchurch New Zealand; ^3^ Department of Surgery and Critical Care University of Otago Christchurch New Zealand

## Abstract

**Background:**

Major trauma recovery extends beyond physical healing, with psychological outcomes and patient experiences influencing long‐term well‐being. This study explored trauma survivors' experiences of follow‐up care and Accident Compensation Corporation (ACC) in New Zealand, and how these relate to psychological distress and social support.

**Methods:**

A retrospective cohort study was conducted at Christchurch Hospital using New Zealand Major Trauma Registry data (May 2016–March 2020, Injury Severity Score ≥ 12). In 2020, eligible patients were invited to complete a follow‐up survey including DSM‐5—aligned screeners for PTSD, anxiety, and depression, questions on follow‐up care and ACC, and a validated social support scale. Associations were assessed with chi‐square and Mann–Whitney *U* tests; free‐text responses underwent thematic analysis.

**Results:**

Of 415 eligible patients, 134 (32.3%) responded. Issues accessing follow‐up care were reported by 20 patients (14.9%), and ACC‐related challenges by 33 (24.6%). Thematic analysis identified insufficient follow‐up, lack of mental health support, communication gaps, and premature return‐to‐work expectations. Psychological morbidity was strongly associated with reported difficulties: patients screening positive for PTSD, anxiety, or depression were 3–5 times more likely to report problems with follow‐up or ACC (all *p* < 0.001). Lower perceived social support was also significantly associated with increased reported difficulties (*p* = 0.01–0.03). Injury severity and ICU admission were not associated.

**Conclusion:**

Mental health symptoms and poor social support were strongly linked to dissatisfaction with trauma follow‐up and ACC processes. Routine psychological screening, improved communication, and coordinated support may enhance recovery outcomes in New Zealand's no‐fault trauma system.

## 1 Introduction

Major trauma is a leading global cause of death and disability [1], with recovery efforts often focused on physical stabilisation; however, growing attention is now given to long‐term mental health impacts such as post‐traumatic stress disorder (PTSD), depression, and anxiety. Numerous studies have documented that a substantial proportion of trauma survivors develop these conditions in the months following injury [2, 3, 4]. Meta‐analytic estimates suggest PTSD affects 20%–30% of trauma survivors, while rates of depression may be higher [4, 5].

Psychological sequelae may significantly impede recovery. Persistent PTSD or depression is associated with poorer quality of life, reduced return to work, and higher healthcare use [3, 6, 7]. ICU admission [5] and injury severity [8] are risk factors for long‐term psychiatric morbidity. Even patients with less severe injuries may struggle with unrecognised mental health issues [2, 9].

Strong social support networks have been identified as protective factors for recovery. Support from family, friends, or peers is consistently associated with lower PTSD and depression scores and faster recovery [9, 10]. Social support has been shown to reduce the need for formal services and mitigate perceived injustice in compensation systems [9].

While the prevalence and clinical predictors of PTSD following major trauma have been well described, considerably less is known about how patients' experiences of post‐discharge care and compensation systems influence psychological recovery. In particular, few studies have examined the relationship between satisfaction with follow‐up services, navigation of no‐fault compensation schemes, and ongoing psychological distress. This is especially relevant in New Zealand, where the universal Accident Compensation Corporation (ACC) system plays a central role in rehabilitation and income support to enable recovery [11]. Patient experiences navigating ACC vary, and challenges such as delays, unclear communication, or return‐to‐work pressure have been shown to influence psychological outcomes [12, 13].

Our research team previously investigated prevalence and predictors of PTSD among major trauma survivors in Christchurch, New Zealand, finding PTSD rates of 18% post injury [8]. Building on that work, this sub‐study aimed to examine patient experiences in post‐hospital trauma care. We aimed to assess satisfaction with follow‐up services, challenges dealing with ACC, and perceived social support. We hypothesised that unmet needs or dissatisfaction in follow‐up care and compensation processes may be associated with higher levels of psychological distress. Identifying such associations may inform improvements to trauma recovery systems.

## 2 Methods

### 2.1 Study Design and Setting

We conducted a single‐centre retrospective cohort study at Christchurch Hospital (Canterbury, New Zealand) [8].

### 2.2 Participants

Eligible patients were those recorded in the New Zealand Major Trauma Registry (NZ‐MTR) who sustained major trauma (Injury Severity Score (ISS) ≥ 12) between May 2016 and March 2020. The NZ‐MTR provided baseline demographic and injury data.

### 2.3 Data Collection

Eligible patients were invited to complete the self‐reported questionnaires. PTSD was measured using the PTSD Checklist for *DSM‐5* [14, 15, 16]. This is a 20‐item self‐report measure that assesses DSM‐5 symptoms of PTSD over the past month according to a 5‐point scale ranging from “not at all (0),” “a little bit (1),” “moderately (2),” “quite a bit (3),” and “extremely (4).” The PTSD questionnaire was tailored so as to emphasise it was about the specific injury [8].

Anxiety and depression were measured using the Hospital Anxiety and Depression Scale [17]. This is a 14‐item self‐report questionnaire used for screening patients in a hospital context for anxiety and depression. The presence and severity of symptoms are rated on a four‐point Likert scale. It omits questions related to symptoms that may be caused by physical illness (e.g., dizziness) and concentrates on psychological aspects of anxiety and depression.

The Multidimensional Scale of Perceived Social Support measured perceived social support [18]. It has 12 items covering family, friends, and significant other. Statements indicative of perceived support (e.g., “I can talk about my problems with my friends”; “There is a special person who is around when I am in need”) are rated on a seven‐point Likert scale (1 = very strongly disagree; 7 = very strongly agree).

Post‐discharge care and ACC experiences were self‐reported via a questionnaire made specifically for this study (Supporting Information 1).

### 2.4 Ethical Considerations

This study was approved by the New Zealand Health and Disability Ethics Committee (Ref: 20/CEN/8/AM03). Participants gave written informed consent. The study adhered to ethical standards for research involving human participants. Māori consultation was undertaken with Te Komiti Whakarite and locality authorisation was given by the Christchurch Hospital Research Office (Ref # 20195).

### 2.5 Statistical Analysis

Quantitative data were analysed using IBM SPSS Statistics (Version 26). Descriptive statistics were used to summarise baseline characteristics. For bivariate analysis, Pearson's chi‐square tests were used for categorical data, and Mann–Whitney *U* tests for continuous data. Statistical significance was set at *p* < 0.05 (two‐tailed). Key grouping variables were based on patient‐reported outcomes: dissatisfaction with follow‐up care and ACC challenges. We explored associations between these outcomes and participant characteristics, including mental health screening status, ISS, ICU admission, gender, and injury mechanism.

### 2.6 Qualitative Analysis

Free‐text responses from the follow‐up survey were analysed using reflexive thematic analysis, following the six‐phase recursive framework proposed by Braun and Clarke [19]. The process began with familiarization of data, involving repeated reading of the survey responses to immerse the researchers in the data. Initial codes were then generated inductively, capturing meanings across the entire dataset without the use of a pre‐existing coding frame. These codes were iteratively grouped to identify candidate themes. Unlike a purely descriptive summary, these themes were developed through a reflexive process, where the researchers critically considered how their own perspectives influenced data interpretation. Themes were then reviewed and refined to ensure they accurately reflected the coded extracts and the dataset as a whole, culminating in the definition and naming of final themes that integrated the nuances of participant experiences of follow‐up support after major injury.

## 3 Results

### 3.1 Patient Characteristics and Injury Factors

A total of 134 of 415 participants returned completed questionnaires (response rate 32.3%). Responders were older than non‐responders (median 55 v 41, *p* = 0.006), there was no difference in sex (*p* = 0.80), and Europeans had the highest response rate (36% European v 9% Māori v 0% Pacific Island v 8% all others, *p* < 0.001). Among responders, the median age was 56 years (IQR 46–67), and 75% were male. Most participants (94%) identified as New Zealand European. The most common injury mechanisms were vehicle‐related trauma (50%), sports (32%), and falls (14%). ICU admission was required in 23% of cases. The median ISS was 16 (IQR 13—Table 1).

**TABLE 1:** Patient baseline demographics and injury factors.

	Category	*n* (%)/Median (IQR)
*Demographics*		
Sex	Female	33 (24.6%)
	Male	101 (75.4%)
Ethnicity	New Zealand European	129 (96.3%)
	Māori	3 (2.2%)
	All others	2 (1.5%)
Age (years)		55 (IQR: 44–62.25)
Years between injury and initial study invitation		2.32 (IQR: 1.96–2.69)
*Injury and Mechanism*		
Mechanism of injury	Vehicle‐related	67 (50.0%)
	Sport‐related	43 (32.1%)
	Fall	19 (14.2%)
	Workplace	6 (4.5%)
	Assault	1 (0.7%)
*Other clinical indicators*		
ICU admission		31 (23.1%)
Alcohol‐related		6 (4.5%)
Injury Severity Score (ISS)		16 (IQR: 13–20.5)
Length of stay (days)		7 (IQR: 4–13)

### 3.2 Qualitative Findings—Follow‐Up Care and ACC

#### 3.2.1 Patient‐Reported Issues With Follow‐Up Care

More than a quarter of participants (26.1%) described significant barriers to follow‐up care. Qualitative responses to the question “Did you have any trouble accessing any other kinds of support after your injury?” were thematically analysed, with the full set of direct quotes presented in Table S2 and a summary of key themes shown in Table 2. Three major themes were identified: inadequate support services, follow‐up assessment and management issues, and navigation barriers. The most common theme, inadequate support services (*n* = 8), reflected a mismatch between the level of assistance participants felt they required and what was provided, including limited rehabilitation input, unreliable home help, and premature return‐to‐work expectations. Follow‐up assessment and management issues (*n* = 4) captured experiences of delayed investigations, inadequate pain management, and a lack of coordinated ongoing care. A smaller group (*n* = 3) described uncertainty about what services were available or how to access them, reflecting navigation barriers within the system. Overall, participants' comments highlighted inconsistent support pathways and a perceived absence of structured follow‐up for trauma survivors once initial hospital care had ended.

**TABLE 2:** Summary thematic analysis of patient‐reported issues with post‐trauma care with example patient quotes.

Did you have any trouble accessing any other kinds of support after your injury?		
Theme	Description	Example participant quotes
Inadequate support services (8 responses)	Participants described a dissonance between the support they felt was required and the support they received.	I was only in hospital for 2 days after the accident and then pretty well bedridden for a couple of weeks and did not feel like reaching out for help. Home help for shaving, removing neck brace etc. was not very reliable.
		When I got back to work everything was hard. It's like you are back at work so their job is done.
Follow up assessments and management issues (6 responses)	Difficulties accessing timely or appropriate follow‐up care after injury.	Difficult to get follow up assessment or advice post discharge from hospital
		It did take over a year and many appointments to get resolution of the issues presented post injury.
Navigation barriers (3 responses)	Challenges understanding, accessing, or initiating appropriate support services post‐injury.	I'm not sure what support I can have.

*Note:* See Table S1 for full list of quotes.

#### 3.2.2 ACC‐Related Issues

Almost half of participants (43.3%) reported experiencing challenges with ACC following their injury. Qualitative responses to the question “Did ACC present you with any challenges after your injury?” were thematically analysed, with the full set of quotes presented in Table S1 and key themes summarised in Table 3. Four major themes were identified: eligibility and compensation disputes, pressure to return to work, ACC case management and process issues, and access or logistical challenges. Eligibility and compensation disputes (*n* = 8) reflected disagreements over injury classification, income calculations, or entitlement to cover—particularly affecting those between jobs or with variable earnings. An equal number (*n* = 8) described feeling pressured to resume work before they were physically or mentally ready. Case management and process issues (*n* = 10) were the most frequently reported concerns, encompassing repeated case‐manager changes, poor communication, and difficulty obtaining recognition for conditions such as PTSD. A smaller group (*n* = 4) highlighted logistical barriers, including travel, accommodation, and coordination of approved services. Collectively, these findings underscore that administrative complexity and inconsistent communication within ACC processes can impede recovery and add to the stress of post‐injury rehabilitation.

**TABLE 3:** Summary thematic analysis of patient‐reported feedback to ACC.

Did ACC present you with any challenges after your injury?		
Theme	Description	Selection of participant quotes
Eligibility and compensation disputes (8 responses)	Disagreements over injury classification, income calculations, or eligibility for compensation.	I had to fight to get compensation after my accident because it was based upon my earnings while I was working part time as a student. Instead on the contract I had signed for the work I was doing at the time.
		I had just left a job and was about to start another when accident happened. Literally was about to start new job but received nothing in compensation from ACC because I wasn't working at the time.
		Minor financial issues relating to secondary income taxes whilst returning to work on light duties. More my fault than theirs to be fair.
Pressure to return to work (8 responses)	Feeling pushed to resume work before being physically or mentally ready.	I felt pressured into getting back into the workforce after having to sell my business after my accident.
		They put me on a work trial that my body couldn't handle
		To begin with they were amazing however towards the end I felt like they didn't listen to me at all, I felt like I had to go back to work way quicker than I should of and I wasn't ready, the lady I felt with was super rude.
ACC case management and process issues (10 responses)	Problems with case manager changes, lack of continuity, delays, or communication issues.	Changing case managers, long convoluted processes, withdrawal of support for rehab before I was ready, ongoing support (3 years post injury) not clear
		Got told I have no case manager. Not forthcoming in offering support services.
		Initial diagnosis of my injury was relatively minor and wasn't amended in ACCs records. This meant there was some obstruction to further treatment as it wasn't clear why I needed more surgery and support. ACC did not want to accept PTSD diagnosis from several GPs and dragged‐out assessment. Also, ACC's structures and systems are a total shambles which makes it hard to communicate with them.
		I was supported during the 8 months following the accident but left to my own devices when back at work and it go so much harder then
Access and logistical challenges (4 responses)	Difficulties with travel arrangements, service coordination, or accessing approved services.	Challenges as a self‐employed earner. Very limited assistance with domestic help following discharge from hospital. This added a lot of unnecessary stress to our home situation.
		Travel and accommodation for treatment was difficult to organise and not always covered.

*Note:* See Table S2 for full list of quotes.

### 3.3 Quantitative Associations

Poorer psychological health was strongly associated with increased reports of ACC‐related challenges and difficulty accessing follow‐up support (Table 4). For all three mental health measures (PTSD, anxiety, and depression), participants above the screening threshold were significantly more likely to report issues than those below the threshold (*p* < 0.001 for all comparisons, *χ* ^2^ tests).

**TABLE 4:** Factors associated with patient‐reported ACC challenges and follow‐up support issues after major trauma.

		ACC challenges			Did you have any trouble accessing any other kinds of support after your injury?		
		Total	*n* (%)	*p*	*n* total	*n* (%)	*p*
Total		134	33 (24.6)	—	129	21 (16.3)	—
Age median split	≤ 55	68	21 (30.9)	0.09	64	16 (25.0)	0.01
	> 55	66	12 (18.2)		65	5 (7.7)	
Gender	Female	33	9 (27.3)	0.68	32	8 (25)	0.12
	Male	101	24 (23.8)		97	13 (13.4)	
ISS median split	≤ 16	68	14 (20.6)	0.27	65	12 (18.5)	0.50
	> 16	66	19 (28.8)		64	9 (14.1)	
GCS in ED	< 14	24	7 (29.2)	0.57	23	5 (21.7)	0.43
	≥ 14	101	26 (23.6)		106	16 (15.1)	
Ethnicity	Māori	3	0 (0)	0.43	3	0	0.60
	NZ European	129	33 (25.6)		124	21 (16.9)	
	Other	2	0 (0)		2	0 (0)	
Required ICU admission	Yes	31	9 (29)	0.52	29	6 (20.7)	0.47
	No	103	24 (23.3)		100	15 (15)	
Vehicle‐related	Yes	67	20 (29.9)	0.16	64	10 (15.6)	0.84
	No	67	13 (19.4)		65	11 (16.9)	
Sport‐related	Yes	43	8 (18.6)	0.27	43	7 (16.3)	1
	No	91	25 (27.5)		86	14 (16.3)	
Fall‐related	Yes	19	5 (26.3)	0.85	112	18 (16.1)	0.87
	No	115	28 (24.3)		17	3 (17.6)	
Workplace‐related	Yes	6	1 (16.7)	0.64	6	0 (0)	0.27
	No	128	32 (25)		123	21 (17.1)	
Assault	Yes	1	1 (100)	0.08	1	0 (0)	0.66
	No	133	32 (24.1)		128	21 (16.4)	
Alcohol involvement	Yes	6	2 (33.3)	0.61	6	1 (16.7)	0.98
	No	128	31 (24.2)		123	20 (16.3)	
Meets PTSD threshold	Yes	23	15 (65.2)	< 0.001	20	9 (45)	< 0.001
	No	111	18 (16.2)		109	12 (11)	
Meets anxiety threshold	Yes	43	20 (46.5)	< 0.001	40	12 (30)	< 0.001
	No	90	12 (13.3)		88	8 (9.1)	
Meets depression threshold	Yes	34	17 (50)	< 0.001	32	13 (40.6)	< 0.001
	No	98	16		95	8 (8.4)	
PTSD or anxiety or depression before injury	Yes	31	6 (19.4)	0.44	30	5 (16.7)	0.95
	No	103	27 (26.2)		99	16 (16.2)	

Participants meeting PTSD threshold were four times more likely to report ACC difficulties (65.2% vs. 16.2%, *p* < 0.001) and five times more likely to report follow‐up problems (45% vs. 9%, *p* < 0.001). Similar differences were observed for anxiety (46.5% vs. 13.3% for ACC, *p* < 0.001; 30% vs. 9.1% for follow‐up, *p* < 0.001) and depression (50% vs. 16% for ACC, *p* < 0.001; 40.6% vs. 8.4% for follow‐up, *p* < 0.001). These represent large and consistent effect sizes, suggesting a link between mental health symptoms and perceived care barriers. In contrast, demographic and injury‐related variables were not significantly associated with either outcome.

Perceived social support emerged as a clear protective factor. Patients who reported stronger support from family, friends, or significant others consistently reported fewer difficulties. Those who reported ACC challenges had significantly lower support scores across all domains (family, friends, and significant others; *p* = 0.02–0.03). Participants who experienced follow‐up problems had significantly lower support from family and significant others (*p* = 0.01–0.02) (Table 5). While the absolute differences were modest (approximately one point on the seven‐point scale), this represents a meaningful perceptual shift in support, consistent with prior Multidimensional Scale of Perceived Social Support research linking similar changes to poorer psychological and functional recovery after trauma [18, 20].

**TABLE 5:** Patient self‐reported social support levels between participants who reported ACC challenges or follow‐up challenges versus those who did not.

	Support type	Median self‐reported social support level (Measured on a scale 1 = very limited support to 7 = very significant support)		*p*
		Yes	No	
Reported ACC challenges	Total	5.17	6.00	0.02
	Significant other	5.75	6.75	0.02
	Family	4.75	6.00	0.02
	Friends	5.00	5.75	0.03
Reported follow‐up challenges	Total	4.58	6.00	0.03
	Significant other	5.00	6.50	0.02
	Family	4.75	6.00	0.01
	Friends	4.75	5.75	0.08

## 4 Discussion

This study explored the long‐term recovery experiences of major trauma survivors in New Zealand, focusing on satisfaction with follow‐up care, interactions with ACC, and associations with psychological outcomes. Key findings emerged: a strong relationship between mental health symptoms and care dissatisfaction, and a protective role of social support.

Patients who screened positive for PTSD, anxiety, or depression were more likely to report dissatisfaction with follow‐up services and difficulties navigating ACC. This may reflect a bi‐directional relationship: inadequate support may exacerbate psychological distress, while pre‐existing distress can magnify the challenges of engaging with health and administrative systems [3, 5, 21]. Our findings align with previous research showing high rates of psychiatric morbidity after trauma—depression in one‐third of orthopaedic trauma survivors and PTSD in up to one‐quarter [4]. In New Zealand, PTSD symptoms have been reported in 12%–17% of injured adults at one‐year post‐injury [2]. Together with evidence that ICU admission independently increases long‐term PTSD risk [5],these results reinforce the need to integrate routine psychological assessment and support into trauma pathways.

Social support clearly buffered patients against system difficulties. Those with stronger support from family, extended family (also called “whānau” in Māori), and peers were less likely to experience problems with ACC or follow‐up care. This is in concordance with evidence from Orlas et al., who found that low social support predicted poorer functional and psychological outcomes after injury across multiple U.S. trauma centres [6]. Similarly, Prang et al. reported that social support improved physical recovery, reduced pain, and in some cases, improved return‐to‐work rates [10]. Evidence also suggests that strong informal support can reduce reliance on formal rehabilitation services [9]. These findings emphasize the importance of involving extended family and community networks in recovery planning and developing peer‐support initiatives within trauma systems.

Experiences with ACC were often challenging, with 43.3% of participants reporting difficulties. The most common issues were inconsistent communication, frequent staff changes, and premature return‐to‐work expectations. International evidence shows that perceived injustice in compensation processes worsens psychological outcomes [22], whereas perceived fairness in no‐fault schemes like ACC is associated with better health [13]. Our results suggest that the delivery of ACC services—particularly case management continuity and communication—may be as important as the entitlements themselves.

Return‐to‐work pressure was a prominent theme. Several participants felt pushed into employment before they felt physically or psychologically ready, echoing findings that premature return to work can worsen PTSD and delay recovery [13, 21, 23]. Expectations should therefore be individualised, aligned with clinical advice, and realistic about job demands. Improving clarity, continuity, and trauma‐informed communication within ACC could substantially reduce the psychological burden of navigating recovery.

### 4.1 Limitations

Ethnicity data were limited, with 96% identifying as New Zealand European and few Māori or Pacific participants, reducing applicability to the wider trauma population. The modest sample size limited statistical power and the response rate of 32.3% will have introduced response bias; indeed older and more European people were more likely to respond. There were perhaps cultural barriers for non‐Europeans, although Māori consultation was undertaken before commencement of this study. The Covid‐19 pandemic occurred after most participants sustained injuries but before their responses, and this may have disrupted responses. Psychological outcomes were based on retrospective self‐report and screening tools rather than formal diagnostic assessment. The median time after injury for study participation was 2.32 years. For some people, they may have had different answers earlier in their post‐injury journey. These limitations should be considered when interpreting the clinical relevance of the findings. Future work should include larger, more representative cohorts and culturally responsive recruitment strategies to strengthen external validity.

### 4.2 Conclusions

This study highlights the hidden challenges many major trauma survivors face in New Zealand after hospital discharge. Difficulties with follow‐up care and ACC processes were strongly linked to symptoms of PTSD, anxiety, and depression, while social support emerged as a key protective factor. These findings reinforce the need for routine psychological screening, better integration of mental health support, and improved communication and continuity in post‐injury services. For ACC, trauma‐informed case management and greater flexibility around return‐to‐work expectations could reduce patient distress. Strengthening formal health services and informal extended family‐ and peer‐based supports may help ensure that trauma recovery is not only physically restorative but also about restoring wellbeing, dignity, and long‐term function.

## Author Contributions

**Duncan Finlayson:** data curation, project administration, writing – review and editing. **Sarah Logan:** data curation, project administration, writing – review and editing, writing – original draft. **Laura R. Joyce:** writing – review and editing, supervision. **Daniel Jemberie:** writing – review and editing, project administration, data curation. **Nikita Quinn:** data curation, project administration, writing – review and editing. **Christopher Wakeman:** writing – review and editing, supervision, conceptualization. **Andrew McCombie:** writing – review and editing, supervision, data curation, project administration, methodology, conceptualization.

## Funding

This work was supported by the University of Otago via a University of Otago Research Grant.

## Ethics Statement

Ethical approval was provided by the New Zealand Health and Disability Ethics Committee (Reference Number 20/CEN/8/AM03).

## Supporting information


**Supporting Information: 1:** Quality of follow‐up questionnaire

**Table S1:** Thematic analysis of patient‐reported issues with post‐trauma care with direct quotes
**Table S2:** Thematic analysis of patient‐reported feedback to ACC with direct quotes

## Data Availability

The data that support the findings of this study are available from the corresponding author upon reasonable request.
