# Supplementary material for: Left Behind After Impact: The Hidden Toll of Trauma Recovery in New Zealand
Source: ANZ J Surg. 2026 May 5;96(9):2353–60. doi: 10.1111/ans.70722 (PMC13602427; doi:10.1111/ans.70722)
Supplement: Supplementary file 1 [file ANS-96-2353-s001.docx]

Supplementary Material 1: Quality of follow-up questionnaire

Please answer these questions in relation to the traumatic injury you experienced.

Did ACC present you with any challenges after your injury?

- No
- Yes (please expand)______________________________________________________________________________________________________________________________________________________________________________________________________________________________________________________________________________________________________

Did you have any trouble accessing other kinds of support after your injury?

- No
- Yes (please expand)______________________________________________________________________________________________________________________________________________________________________________________________________________________________________________________________________________________________________

Do you feel you had a head injury missed during treatment and follow up?

- No
- Yes (please expand)______________________________________________________________________________________________________________________________________________________________________________________________________________________________________________________________________________________________________

How much do you agree with the following statement? (tick one answer)

*“My treatment needs were met following my traumatic injury”*

| ¨ | ¨ | ¨ | ¨ | ¨ |
| --- | --- | --- | --- | --- |
| Strongly disagree | Disagree | Do not agree or disagree | Agree | Strongly agree |

Do you have any other comments regarding treatment and follow up?

________________________________________________________________________________________________________________________________________________________________________________________________________________________________________________________________________________________________________________________________________
