# Supplementary material for: Left Behind After Impact: The Hidden Toll of Trauma Recovery in New Zealand
Source: ANZ J Surg. 2026 May 5;96(9):2353–60. doi: 10.1111/ans.70722 (PMC13602427; doi:10.1111/ans.70722)
Supplement: Supplementary file 2 [file ANS-96-2353-s002.docx]

***Supplementary Table 1: Thematic analysis of patient-reported issues with post-trauma care with direct quotes.***

| Did you have any trouble accessing any other kinds of support after your injury? | | |
| --- | --- | --- |
| **Theme** | **Description** | **Participant Quote** |
| Inadequate support services  (8 responses) | Participants described a dissonance between the support they felt was required and the support they received. | *After my injury I was given 6 weeks one day a week to learn to live life with the sight of one eye* |
|  |  | *I was only in hospital for 2 days after the accident and then pretty well bedridden for a couple of weeks and did not feel like reaching out for help* |
|  |  | *Home help for shaving, removing neck brace etc was not very reliable.* |
|  |  | *Support person for pool therapy. Travel and holiday support difficult to get.* |
|  |  | *More support from the occupational therapy people. Felt like they were dossing me back to work too soon.* |
|  |  | *Nothing was offered by hospital or ACC. I went and got a medical sign off to uplift my MNZ [motorcycle NZ] licence to enable me to race again.* |
|  |  | *Physio was left to me* |
|  |  | *When I got back to work everything was hard. It's like you are back at work so their job is done.* |
| Follow up assessments and management issues  (4 responses) | Difficulties accessing timely or appropriate follow-up care after injury. | *Issues with getting nerve conduction study done as no clinicians were available to do it. Aside from that no real issues.* |
|  |  | *Still go this day I am in pain and have issues with my back and hip. I am STILL waiting for surgery which no one has ever got in contact with me regarding. I have only had one follow up appointment and apart from that they have left me to it* |
|  |  | *Difficult to get follow up assessment or advice post discharge from hospital* |
|  |  | *It did take over a year and many appointments to get resolution of the issues presented post injury.* |
|  |  | *No pain management offered.* |
|  |  | *My GP provided almost nothing helpful or supportive, i.e. nothing for physio or counselling offered to me. Not listening or caring enough and mostly doing paperwork for ACC and writing prescriptions.* |
| Navigation barriers  (3 responses) | Challenges understanding, accessing, or initiating appropriate support services post-injury. | *I'm not sure what support I can have.* |

***Supplementary Table 2: Thematic analysis of patient-reported feedback to ACC with direct quotes***

| **Theme** | **Description** | **Participant Quote** |
| --- | --- | --- |
| **Eligibility and compensation disputes**  (8 responses) | Disagreements over injury classification, income calculations, or eligibility for compensation. | *They deemed it not a work injury although I was white baiting as a job at the time and had done for the previous 3 years* |
|  |  | *I had to fight to get compensation after my accident because it was based upon my earnings while I was working part time as a student. Instead on the contract I had signed for the work I was doing at the time.* |
|  |  | *Would not pay for loss of wages* |
|  |  | *I had just left a job and was about to start another when accident happened. Literally was about to start new job but received nothing in compensation from ACC because I wasn't working at the time.* |
|  |  | *Weekly compensation amount wasn't based upon previous 12 months' income but the last 3 months. Different rules for different people; e.g., taxis for some but we had to catch buses from airport to Burwood (this is one of many examples)* |
|  |  | *Financially* |
|  |  | *Minor financial issues relating to secondary income taxes whilst returning to work on light duties. More my fault than theirs to be fair.* |
|  |  | *For the majority ACC has been very helpful. Although, many costs we had thought would be covered were not or only a small portion was. Thankfully, we are in a financial position that we could afford what I needed. If we were not, I would not know what we would have done.* |
| **Pressure to return to work**  (8 responses) | Feeling pushed to resume work before being physically or mentally ready. | *"Forced" me back to work before my surgeon, my psychologist, and especially I felt ready to be there* |
|  |  | *At the time I was an employed wool handler, working a very physical job in the shearing sheds. They expected me to return to work part time on 'light duties'. 'Light duties' does not exist in the shearing sheds which they couldn't seem to comprehend.* |
|  |  | *Early return to work* |
|  |  | *I felt pressured into getting back into the workforce after having to sell my business after my accident.* |
|  |  | *Sent to an employment agency within 3 months of leaving hospital to look for work even though I already had a job to go back to and felt pushed to see the ACC doctor. ACC case manager via phone was a trigger for absolute panic.* |
|  |  | *They put me on a work trial that my body couldn't handle* |
|  |  | *To begin with they were amazing however towards the end I felt like they didn't listen to me at all, I felt like I had to go back to work way quicker than I should of and I wasn't ready, the lady I felt with was super rude.* |
|  |  | *Mostly great but contacted my employer stating I would be ready to work when I still had an operation to go.* |
| **ACC case management and process issues**  (10 responses) | Problems with case manager changes, lack of continuity, delays, or communication issues. | *changing case managers, long convoluted processes, withdrawal of support for rehab before I was ready, ongoing support (3 years post injury) not clear* |
|  |  | *Had issues with seeking support with rehab and compensation-trying and tried with only barriers* |
|  |  | *Got told I have no case manager. Not forthcoming in offering support services.* |
|  |  | *When trying to get income support… dealing with a different employee each time it felt a case manager left. I also had a head injury so thankfully my lovely husband stepped in and helped sort.* |
|  |  | *only criticism would be time delays between appointments which left me unable to plan ahead but our friend covid may have had a large part to play in this* |
|  |  | *Communicating with them in first 24 hours. I not well enough to do so but wouldn't talk to my wife.* |
|  |  | *Initial diagnosis of my injury was relatively minor and wasn't amended in ACCs records. This meant there was some obstruction to further treatment as it wasn't clear why I needed more surgery and support. ACC did not want to accept PTSD diagnosis from several GPs and dragged-out assessment. Also, ACC's structures and systems are a total shambles which makes it hard to communicate with them.* |
|  |  | *Use to work for them. Very aggressive organisation* |
|  |  | *I was supported during the 8 months following the accident but left to my own devices when back at work and it go so much harder then* |
|  |  | *ACC sent an experienced occupational therapist to my employer to put together a return-to-work program. My employer bullied them into the program that they wanted to create for me which was to get me back to work full time as quickly as possible. This has caused a lot of issues, ongoing physical discomfort etc because of this. It hindered my rehabilitation, and it wasn't until a more experienced occupational therapist came into the picture and put her foot down with my employer to things start getting better* |
| **Access and logistical challenges**  (4 responses) | Difficulties with travel arrangements, service coordination, or accessing approved services. | *Challenges as a self-employed earner. Very limited assistance with domestic help following discharge from hospital. This added a lot of unnecessary stress to our home situation.* |
|  |  | *Except ACC wouldn't pay for flights home to North Island after [a family member] got discharged from hospital.* |
|  |  | *Travel and accommodation for treatment was difficult to organise and not always covered.* |
|  |  | *I think ACC sat back waited for other to take the lead* |
